# Nutritional Profile Changes in an Insect–Fungus Complex of *Antheraea pernyi* Pupa Infected by *Samsoniella hepiali*

**DOI:** 10.3390/foods12142796

**Published:** 2023-07-23

**Authors:** Shengchao Wang, Yun Meng, Dun Wang

**Affiliations:** Institute of Entomology, Northwest A&F University, Xianyang 712100, China; shengchaowang1999@foxmail.com (S.W.); my872187375@foxmail.com (Y.M.)

**Keywords:** *Antherea pernyi* pupa, *Samsoniella hepiali*, insect fungus complex, nutritional profile changes, protein, fat, polysaccharide, chitin, cordycepin

## Abstract

Historically, some edible insects have been processed into a complex of insect and fungus, such as *Antherea pernyi* and *Samsoniella hepiali*. Until now, the dynamics of the nutritional changes due to this infection were unclear. This study reveals the dynamic changes in nutritional components of *Antherea pernyi* pupa after infection with *Samsoniella hepiali* at post-infection time points of 0 d, 10 d, 20 d, and 30 d. The dynamic analysis of the components at different post-infection times showed that the content of polysaccharides and cordycepin increased with time while the content of fats and chitin decreased. The content of proteins showed a trend of decreasing at the beginning and then increasing. The essential amino acids (EAAs) decreased at the beginning and then increased, and non-essential amino acids (NEAA) changed similarly. The essential amino acid index showed a slight continuous decrease. Although the crude fat decreased dramatically due to the infection, from a value of 30.75% to 7.2%, the infection of *S*. *hepiali* produced five new fatty acids (14-methyl-pentadecanoic acid, docosanoic acid, succinic acid, arachidonic acid, and myristic acid) while the content of the seven fatty acids was greatly reduced after infection. Therefore, after being infected by *S*. *hepiali* and combined with it, the nutritional profile of *A pernyi* pupa was changed significantly and there were different characteristics at different infection stages. The above findings provide scientifically fundamental data to understand the nutritional value of the insect–fungus complex as human food and animal feed.

## 1. Introduction

Due to its abundant source of high-quality nutrition, the use of insects as both human food and animal feed has emerged as a mainstream trend, particularly in light of the increasing challenges of globalization [1,2]. The United Nations Food and Agriculture Organization (FAO) mentions that two billion people worldwide eat insects. Although the source of this statistic is unscientific and probably exaggerated [3], it does reflect, to some extent, the existence of a large amount of insect consumption in the world. Emerging foods processed with insects are both delicious and nutritious, and their edible value has been recognized by the European Union [4]. Much research has been performed on insects as food in high-income countries, including surveys on the acceptance of edible insects and studies on their nutritional value. Fischer believes that insects as food should gradually shift from “acceptance” to “desirability”, but there is still a long way to go before this transition is truly achieved [5]. The development of edible insects is still limited [6]. Fortunately, there are ways we can promote the development of edible insects. One method is to produce a mushroom-like complex of an edible insect infected by a medicinal fungal pathogen.

In China, the Tussah (*Antherea pernyi*) pupa is a classic edible insect, rich in various nutrients that are of great benefit to human beings [7,8]. After cooking and processing, the Tussah pupae becomes a delicious dish with good color, smell, and taste. Meanwhile, the nutritional or medicinal value of medicinal fungi have been historically explored and recognized by the public. The so-called “Himalayan Gold” *Ophiocordyceps sinensis* is a representative [9]. Since ancient times, *O. sinensis* has been actively discovered and used as a valuable traditional Chinese medicine material, due to its excellent medical and prophylactic functions in health care and disease prevention [10]. However, due to the significant demand from the market and low reproduction rate, *O. sinens* has been overexploited, and its habitat has been greatly damaged [11]. To address this situation, people need to explore alternatives to replace *O. sinensis*. *Paecilomyces hepiali* (the new species name is *Samsoniella hepiali*) was isolated from natural *O. sinensis* [12,13]. This fungus has been added to the list of fungus strains used as health food, which was promulgated by the China Food and Drug Administration [14]. The various medicinal properties of *S. hepiali* are well recognized. Studies have revealed that *S. hepiali* is rich in polysaccharides, cordycepin, amino acids, nucleosides, organic acids, and other nutrients [15]. Because of the presence of these nutritious and active substances, *S. hepiali* has a variety of pharmacological functions in humans, such as anti-tumor [16,17,18,19], anti-oxidant [20,21], anti-inflammatory [22,23], anti-bacterial [24,25], and anti-fatigue [26,27,28] functions.

Enriching the processing and consumption of forms of edible insects could improve the overall acceptance of edible insects, especially when combined with high-value foods that are already recognized and enjoyed by the public. The mushroom-like complex of Tussah pupa infected by *S. hepiali* has the potential to attract a wider range of consumers. This process could also be extended to other edible insects and medicinal fungi. Apparently, *S. hepiali* absorbs nutrients from the Tussah pupa and converts them into new substances during the infection. In addition, medicinal fungi can enrich the taste of edible insects after infection. The changes in some nutrients of Tussah pupa infected by *Cordyceps militaris* were prefiously analyzed [29]; however, there is no any reference to address the changes in nutrients in the Tussah pupa infected by *S. hepiali*. In this study, the dynamic changes in some nutrients in the pupa of *A. pernyi* infected with *Samsoniella hepiali* were studied with a time course after infection.

## 2. Materials and Methods

### 2.1. Preliminary Preparation

The strain of *Samsoniella hepiali* (NCBI login number:MW391718) was maintained by the Lab of Insect Related Resource (Northwest A&F University, Yangling, China). *A. pernyi* pupae were purchased from the breeding base online, then the healthy and energetic individuals were selected for experiment. All chemical reagents involved in the experiments were purchased from Yuanye Biotechnology Co., Ltd. (Shanghai, China).

The strain was cultured on PDA at 26 °C for 6 days. The PDA (liquid medium) was composed of the following (g/L): yeast extract (5), sucrose (20), wheat bran (10), peptone (5), KH_2_PO_4_ (2.4), and MgSO_4_ (1.2). The hyphae were removed and placed into a 250 mL conical flask containing 100 mL of liquid medium, and cultured at 25 °C for 5 d. Fresh live Tussah pupae were sterilized with 75% alcohol and then inoculated with 0.2 mL of the liquid strain into each one. Pupae were then placed in the dark at 15 °C for 7 days until they hardened. Then, the Tussah pupae were transferred to a light culture. The culture temperature was 22 °C and the light intensity was about 500 lx. During the cultivation period (30 d), sufficient pupae were collected at four different time points (0 d, 10 d, 20 d, 30 d) and then subjected to freeze-drying and crushing treatment. All crushed dry powder was stored in a refrigerator at −20 °C.

### 2.2. Polysaccharide Determination

A sulfate-phenol method was used to determine polysaccharides [30]. Samples were extracted with 80% ethanol for 30 min and ultrasonic extraction to remove impurities. After ultrasonication, the supernatant was discarded and the remaining solid was boiled in boiling water at 100 °C for 3 h to extract polysaccharides. The filtrate was filtered through a 0.22 mm filter membrane. Standard solutions were prepared at different concentrations (20, 40, 60, 80, and 100 mg/L) using glucose standards, and standard curves were plotted to determine the polysaccharide content [31].

### 2.3. Crude Protein Determination

The Kjeldahl method was used for determine the crude protein, and the nitrogen protein conversion factor was 5.41 [32].

### 2.4. Chitin Content Determination

Chitin was extracted by Wang’s method [33]. The samples were treated with 3% hydrochloric acid for 24 h to remove mineral impurities. After hydrochloric acid treatment, the samples were soaked in 10% NaOH at 100 °C for 5 h to remove protein and fat. Finally, the chitin was dehydrated and weighed. 

### 2.5. Crude Fat Determination

The classical Soxhlet extraction method was used for the determination of crude fat. The filter paper was cut into 8 cm × 8 cm and folded into a paper packet without sealing on one side, and then the filter paper packet was weighed (a). The filter paper packet with about 3 g of samples were then weighed (b); (b − a) is the weight of the sample. We then set up a Soxhlet extractor and performed a Soxhlet extraction on the sample. After extraction, we removed the filter paper pack with long tweezers and allowed the ether to evaporate in a ventilated place. After the ether had evaporated, the filter paper bag was dried and weighed (c). Crude fat content = (b − c)/(b − a) × 100%. We placed the extracted fatty acids in the refrigerator at −20 °C.

### 2.6. Fatty Acid Determination

Samples of 2.0 mg of fat were accurately weighed and dissolved in 1.5 mL of methanol; then, 60 μL of methyl acetate was added, and it was shaken and allowed to stand for 10 min. Then we added 100 μL of 27 mg/mL sodium methoxide solution and allowed it to stand for 20 min. This was followed by 60 μL of saturated oxalic acid, then shaken well and allowed to stand for 2 minutes in a −20 °C refrigerator [34]. Finally, the fatty acid composition was determined by gas chromatography (DB-FFAP capillary column, Agilent Company, Santa Clara, CA, USA).

### 2.7. Amino Acid Determination

The samples were pre-treated for 22 h with HCl (6 mol/L) acid at 110 °C; then, levels of cysteine and tryptophan were determined by formic acid oxidation and alkali hydrolysis, respectively [35,36]. The hydrolysate was dehydrated under a vacuum and dissolved with sodium citrate (pH = 2.2) and then filtered with a 0.22 mm microporous membrane filter. Amino acid content was determined by an amino acid analyzer (Beckman 121 MB, Beckman Kurt company, Brea, CA, USA). The Essential Amino Acid Index (EAAI) of the samples was calculated using Oser’s method, and the official data from the FAO and USDA (https://fdc.nal.usda.gov//fdc-app.html#/food-details/748967/nutrients, accessed on 26 March 2023) were used as the basis for subsequent amino acid analysis [37,38].

### 2.8. Cordycepin Determination

The samples were extracted by deionized water using ultrasonic extraction for 0.5 h, and filtered through a 0.22 mm membrane prior to injection into the high-performance liquid chromatography (HPLC) system [30]. Standard samples of cordycepin (0, 5.0, 10.0, 20.0, 50.0 mg/L) were prepared in advance and the standard curves were plotted after HPLC analysis.

### 2.9. Statistical Analysis

All experiments were performed in triplicate and expressed as mean ± SD. Multi group comparisons of the means were carried out by a one-way analysis ANOVA test using SPSS (version 22) with Tukey’s HSD tests. The graphs in this article were drawn using Origin (version 9.6).

## 3. Results and Discussion

### 3.1. The Tissue Changes of the Infected Pupa

**Figure 1:**
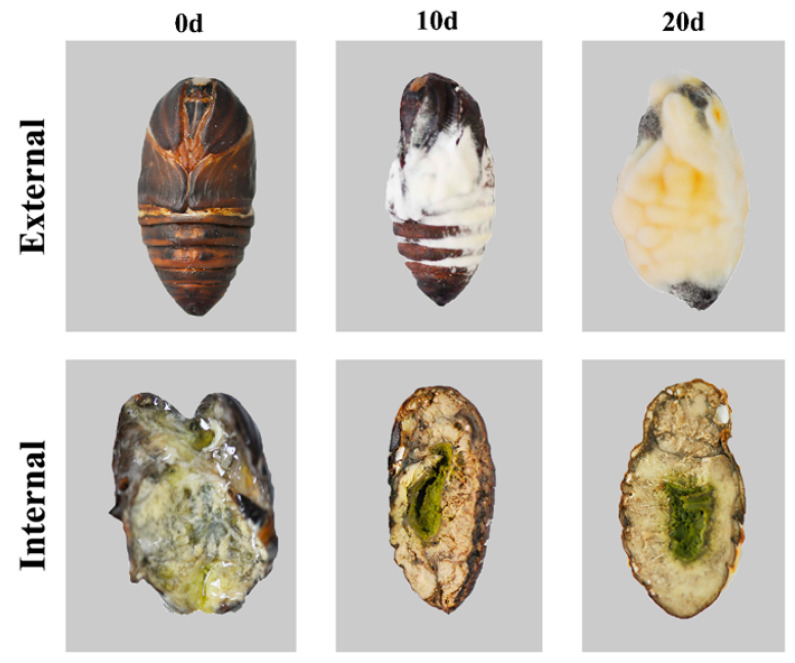
External and interior tissue change of the fungus-infected Tussah pupa.

The tissue of the pupa infected by *S. hepiali* was thoroughly changed (Figure 1). The external surface of the pupa first appeared with a few white-colored mycelia, and as time passed, the mycelia on the surface of the pupa further grew and the color gradually changed to yellow. The internal tissue was transformed into a solid and yellow-brown hard body, and the mycelia gradually replaced more and more areas of the tissue. Additionally, the water content decreased to a visually evident degree.

### 3.2. Polysaccharide Analysis

**Figure 2:**
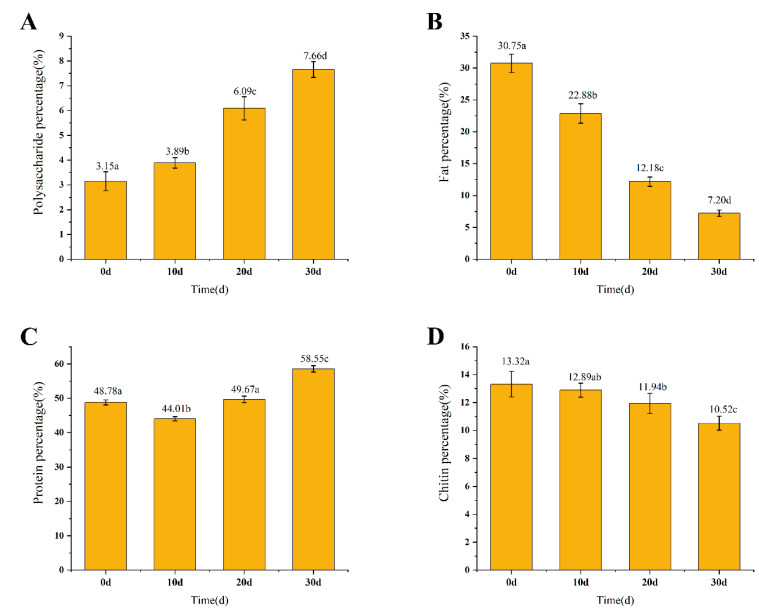
Nutritional dynamics of insect fungus complex. (**A**) Polysaccharide content. (**B**) Fat content. (**C**) Protein content. (**D**) Chitin content. Data are presented as the mean ± SD. Means with different superscripts in the column differ significantly (*p* ≤ 0.05). n = 3.

The dynamic changes in polysaccharide are shown in Figure 2A. The content of polysaccharide in the pupa increases continuously with the prolongation of infection time, from 3.15% at the beginning to 7.66% at the end. This indicates that during the infection process, *S. hepiali* transforms part of the organic substances in the pupa into *S. hepiali* polysaccharides (SPs). As for SP, there have been many studies demonstrating its potential anti-diabetic nephropathy, anti-fatigue, hypoglycemic, anti-depressant, and anti-nociceptive properties [23,27,39,40]. A recent study on db/db mice showed that SP has pharmacological functions such as hypoglycemic, hypolipidemic, anti-diabetic, and anti-nephritic functions; the mechanism underlying this effect may be related to the Nrf2-meadited NF-κB signaling pathway in the mice [41]. The structure of *O. sinens* polysaccharide (OP) and its multiple pharmacological activities have been discovered and published, and Yuan et al. summarized these [42]. Unfortunately, as a fungus isolated from *O. sinens*, there is still limited research conducted on SP internationally, and further exploration and verification are needed to uncover and validate its potential pharmacologic functions in humans.

### 3.3. Crude Fat and Fatty Acid Analysis

The dynamic changes in crude fat are shown in Figure 2B. The crude fat content continued to decrease over the course of infection, with values of 30.75% (0 d), 22.88% (10 d), 12.18% (20 d), and 7.20% (30 d), respectively. The steady decrease in crude fat content suggests that during the infection process, the fat content of the pupa is likely to be consumed in large quantities for the growth of the fungus and the transformation of other substances. However, the variation in fatty acid composition indicates that fat is not simply a consumed item in this process.

The results of fatty acid detection are shown in Table 1. A total of seven fatty acids were detected in *A. pernyi* pupa (0 d), including three SFAs (saturated fatty acids) and four UFAs (unsaturated fatty acids), and the content of these seven fatty acids decreased continuously during the infection process. Interestingly, the infection of *S. hepiali* has greatly reduced the fat content of the pupa, but expanded the variety of fatty acids. Five new fatty acids were produced during the infection process, namely 14-methyl-pentadecanoic acid, docosanoic acid, succinic acid, arachidonic acid, and myristic acid. This means that the richness of different fatty acids of *A. pernyi* is enhanced and this fatty acid variety would be a benefit for consumers who wished to reduce their saturated fatty acids consumption and increase functional fatty acids. For example, the succinic acid is a dicarboxylic acid, also known as butanedioic acid, with the chemical formula C_4_H_6_O_4_. In 1546, Georgius Agricola first purified succinic acid from amber by isolation and then succinic acid was fermented by various microorganisms and used in the food and pharmaceutical industries [43]. Gamage et al. [24] detected succinic acid from the extracts of *S. hepiali* mycelia and found that the extracts may have bactericidal activity in the gastrointestinal tract. Succinic acid is involved in multiple biochemical pathways, can be produced by a variety of microorganisms, and is essential for human energy metabolism as an important intermediate in the tricarboxylic acid (TCA) cycle [44]. Arachidonic acid is a polyunsaturated fatty acid that is a crucial component of biological membranes, prostaglandins, and other arachidonic acids, with the molecular formula C_20_H_32_O_2_. As an inhibitor of immunity, arachidonic acid has antithrombotic and chemotactic effects [45,46]. Non-oxidized derivatives of arachidonic acid are essential components of stress response and mood regulation [47]. For humans, arachidonic acid is an essential fatty acid, and a long-term deficiency in arachidonic acid can lead to serious consequences. Myristic acid is widely distributed in the fats of plants and animals, including common human foods such as nutmeg, and is often used as a flavoring ingredient in the food industry [48]. In investigating whether lauric or myristic acid prevented testosterone-induced prostate hyperplasia in rats, Babu et al. [49] found that lauric/myristic acid had a significant inhibitory effect on prostate hypertrophy and protection of prostate tissue structure, implying that myristic acid had potential medical value. In general, the *S. hepiali* infection has reduced the fat content of the pupa considerably, but made the fatty acid profile more varied.

### 3.4. Crude Protein and Amino Acid Analysis

The dynamic changes in crude protein are shown in Figure 2C. Unlike other components in this study, the change in protein content showed a turning point at 10 d, with a trend of initially decreasing and then increasing (48.78%, 44.01%, 49.67%, and 58.55%, respectively). This trend may indicate that during the process of *S. hepiali* infection, the conversion of nutritional substances is not a simple and unidirectional process, and the actual internal changes are more complex than expected.

The results of amino acid detection are shown in Table 2. A total of 18 amino acids were detected in *A*. *pernyi* pupa (0 d), including 10 EAA (essential amino acids) and 8 NEAA (non-essential amino acids), and the types of amino acids in the protein did not change during the infection process. EAA decreased at the beginning and then increased (20.35%, 17.55%, 17.79%, and 19.51%, respectively, at different time points), and NEAA changed similarly (28.53%, 27.42%, 30.74%, and 36.05%, respectively, at different time points). EAAI shows a continuous slight decrease (0.98, 0.97, 0.95, and 0.92, respectively). The infection time corresponding to the highest value of a single NEAA are as follows: aspartic (30 d), serine (20 d), glutamate (30 d), proline (30 d), glycine (30 d), alanine (30 d), cysteine (0 d), tyrosine (0 d), histidine (0 d), and arginine (30 d); and that of EAA are as follows: threonine (30 d), valine (30 d), methionine (0 d), isoleucine (30 d), leucine (30 d), phenylalanine (0 d), lysine (0 d), and tryptophan (0 d).

**Figure 3:**
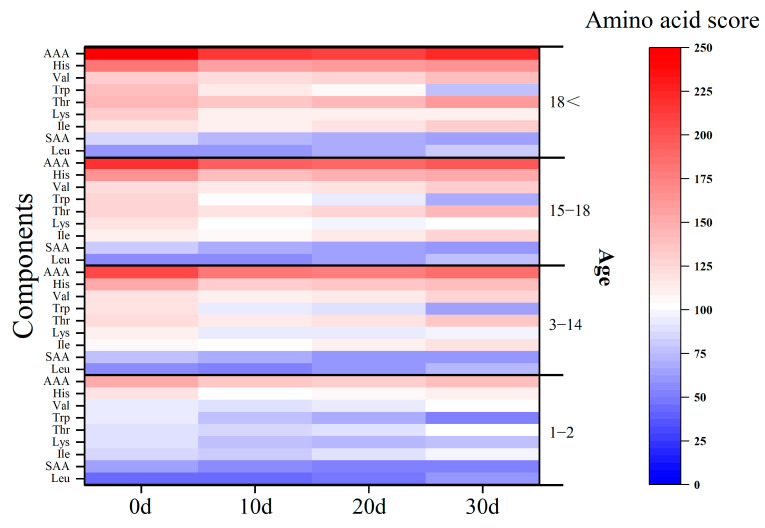
Amino acid scores at the different infected stages of the insect–fungus complex for infants, children, adolescents, and adults.

Except for the initial stage, the protein content of the insect–fungus complex generally increases, and the protein content becomes more abundant. However, if we consider the amino acid composition, the discussion becomes more complex. During a month-long infection, the increase in total protein content is primarily due to the increase in NEAA, while the relative content of EEA, which is more important for humans, has decreased compared to the initial stage. It can be found that the highest values of a different single amino acid mainly occurred at 0 d and 30 d after infection, indicating that the infection of *S. hepiali* caused the continuous increase in the content of some amino acids while the content of some other amino acids continuously decreased; this feature can provide dietary guidance for people with different amino acid needs. The value of the EAAI (essential amino acid index) reflects the reasonableness of the composition of essential amino acids in foods. The closer the EAAI value to 1, the higher the nutritional value of protein in foods [37]. Therefore, from the perspective of the EAAI, the infection of *S. hepiali* slightly reduces the protein quality of the pupa. In addition, amino acid scores were obtained for pupae at different stages of infection based on the amino acid scoring pattern for infants, children, adolescents, and adults from the 2007 WHO/FAO/UNU report [38]. It is clear that both the pupae and the insect fungus complex are more suitable for adult consumption, with the *S. hepiali* infection allowing the scores of each amino acid category to fluctuate (Figure 3). After 10 days of infection, the total amount of protein in the pupa increases, but the quality of the protein slightly declines.

### 3.5. Chitin Analysis

The dynamic changes in chitin are shown in Figure 2D. The content of chitin decreased continuously with increasing *S. hepiali* invasion time, which was 13.32% (0 d), 12.89% (10 d), 11.94% (20 d), and 10.52% (30 d). Chitinase can be produced by various organisms such as bacteria, fungi, viruses, insects, plants, and animals [50]. The decrease in chitin may indicate that *S. hepiali* secreted chitinase to digest the chitin in the cuticle of the pupa when piercing through the cuticle to emerge. Chitin is the main component of the arthropod’s exoskeleton and is highly valued for its bioactivity; these bioactivity characteristics include immune stimulation, growth promotion of beneficial bacteria, and inhibition of the growth and activity of pathogenic bacteria [51,52,53]. As part of the exoskeleton of insects, chitin is a biopolymer that mammals lack. Therefore, chitin is an easy target for the mammalian immune system; in vivo experiments in fish, pigs, and poultry showed that proper consumption of chitin strengthens the immune system and prevents pathogen reproduction; chitin also has hypolipidemic and antioxidant properties and positively regulates the host’s intestinal bacterial community [54]. Chitin cannot be directly absorbed by the human body, but it may be used as an alternative to antibiotics to prevent the potential bacterial infectious risks in animal feed, which can then reduce human health risks [55,56]. The decrease in chitin means a weakening of the above-mentioned functions of insects as feed, but its inability to be directly absorbed also indicates that when humans consume the insect–fungus complex as food directly, they will face lower digestive pressures.

### 3.6. Cordycepin Analysis

**Figure 4:**
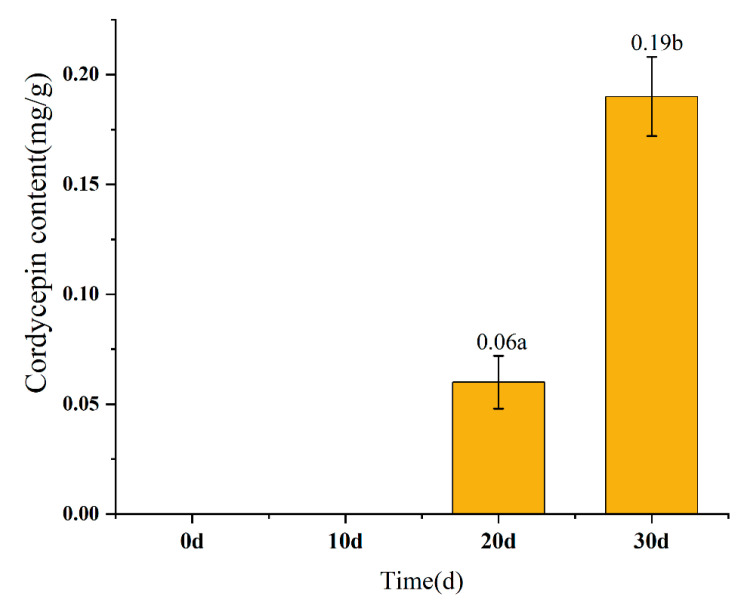
The dynamic changes in cordycepin quantity. Data are presented as the mean ± SD. Means with different superscripts in column differ significantly (*p* ≤ 0.05). *n* = 3.

The dynamic changes in cordycepin are shown in Figure 4. Cordycepin was not detected in the pupa at 10 d and 20 d. At 20 d, the amount of cordycepin in the pupa was 0.06 mg/g, and at 30 d, this value increased to 0.19 mg/g. Cordycepin is an important natural nucleoside compound isolated from *C. militaris* [57]. Several studies have shown that cordycepin and its derivatives (such as pentostatin) exhibit a wide range of health-promoting effects in humans, including but not limited to anti-bacterial activity, anti-cancer effects, anti-oxidant effects, and anti-inflammatory effects [58,59,60,61]. Mao et al. [62] found that the samples of *O. sinens* from Naqi City, Tibet province, China, contained 0.3 mg/g of cordycepin. However, Ge et al. [63] measured the cordycepin content in the fermented mycelium of *S. hepiali* and found it to be 0.44 mg/g, almost 15 times higher than in *O. sinens*. The content of cordycepin in this study reached a maximum of 0.19 mg/g after 30 days of cultivation, which still represents a significant gap from the *S. hepiali* fermentation mycelium. The production of cordycepin makes the pupa more nutritionally valuable, but the concentration is still not satisfactory. Perhaps it can be achieved by prolonging the cultivation time to meet higher content requirements; at this time, it is also necessary to consider the changes in other nutritional substances. Kaushik et al. [64] found that appropriate supplementation of hypoxanthine, adenosine, glycine, glutamine, aspartic acid, NAA(1-naphthaleneacetic acid), IAA(3-indoleacetic acid), and thiamine (B1) could increase cordycepin production in *O. sinensis*. This finding provides an important possibility for improving the culture condition by adding the above-mentioned substances to increase the rate of cordycepin production in the insect–fungus complex.

## 4. Conclusions

In this study, with the prolongation of infection time, several important nutritional components in Tussah pupa increased or decreased significantly, including polysaccharides, cordycepin, and proteins, as well as chitin and fats. Additionally, the detection of amino acids and fatty acids indicates that the transformation of materials during the infection process is diverse and complex. For example, while the total amount of fats significantly decreased, the variety of fat acids became more abundant. Without specific requirements or unified standards, it is difficult to make a simple conclusion about the comprehensive nutritional value of pupa infected at different times. Our research results have different guiding values for different nutritional or taste needs of people. To reveal the benefits or disadvantages of this new insect–fungus complex as human food and animal feed, additional studies are needed, including expanding the scope of component detection and further extending the duration of infection.

## Figures and Tables

**Table 1:** The content of fatty acids at different infection times (%, dry matter basis).

Item		Time(d)			
		0 d	10 d	20 d	30 d
**SFA (%)**	Palmitic acid	6.22 ± 0.08 ^a^	4.58 ± 0.09 ^b^	2.44 ± 0.06 ^c^	1.45 ± 0.03 ^d^
	Stearic acid	0.62 ± 0.04 ^a^	0.69 ± 0.03 ^a^	0.49 ± 0.04 ^b^	0.37 ± 0.05 ^c^
	Heptadecanoic acid	0.19 ± 0.03 ^a^	0.12 ± 0.01 ^b^	0.05 ± 0.01 ^c^	0.03 ± 0.00 ^c^
	14-methyl-pentadecanoic acid	0.00 ± 0.00 ^a^	0.03 ± 0.00 ^b^	0.05 ± 0.00 ^c^	0.08 ± 0.01 ^d^
	docosanoic acid	0.00 ± 0.00 ^a^	0.04 ± 0.01 ^b^	0.03 ± 0.01 ^b^	0.02 ± 0.00 ^b^
	succinic acid	0.00 ± 0.00 ^a^	0.07 ± 0.02 ^b^	0.08 ± 0.01 ^b^	0.08 ± 0.01 ^b^
	Subtotal	7.04 ± 0.11 ^a^	5.53 ± 0.14 ^b^	3.13 ± 0.12 ^c^	2.03 ± 0.06 ^d^
**UFA (%)**	Palmitoleic acid	1.48 ± 0.04 ^a^	1.03 ± 0.03 ^b^	0.49 ± 0.01 ^c^	0.28 ± 0.02 ^d^
	Oleic acid	9.64 ± 0.15 ^a^	6.98 ± 0.11 ^b^	3.59 ± 0.04 ^c^	1.75 ± 0.03 ^d^
	Linoleic acid	2.17 ± 0.05 ^a^	2.43 ± 0.04 ^b^	1.89 ± 0.05 ^c^	1.75 ± 0.05 ^d^
	α-linolenic acid	10.63 ± 0.03 ^a^	6.75 ± 0.07 ^b^	2.82 ± 0.03 ^c^	1.18 ± 0.04 ^d^
	Arachidonic acid	0.00 ± 0.00 ^a^	0.03 ± 0.01 ^b^	0.04 ± 0.00 ^b^	0.06 ± 0.01 ^c^
	Myristic acid	0.00 ± 0.00 ^a^	0.09 ± 0.02 ^b^	0.07 ± 0.01 ^bc^	0.05 ± 0.01 ^c^
	Subtotal	23.92 ± 0.21 ^a^	17.33 ± 0.14 ^b^	8.90 ± 0.12 ^c^	5.04 ± 0.08 ^d^
**Total (%)**		30.96 ± 0.31 ^a^	22.84 ± 0.24 ^b^	12.04 ± 0.15 ^c^	7.09 ± 0.14 ^d^
**UFA/Total**		0.77	0.76	0.74	0.72

SFA, saturated fatty acid; UFA, unsaturated fatty acid. Data are presented as the mean ± SD. Means with different superscripts in column differ significantly (*p* ≤ 0.05). *n* = 3.

**Table 2:** The content of amino acids at different infection times (%, dry matter basis).

Item		Time (d)			
		0 d	10 d	20 d	30 d
**NEEA (%)**	Aspartic	5.53 ± 0.06 ^a^	5.43 ± 0.06 ^a^	6.13 ± 0.09 ^b^	7.51 ± 0.13 ^c^
	Serine	1.50 ± 0.07 ^a^	1.48 ± 0.03 ^ab^	1.69 ± 0.04 ^c^	1.46 ± 0.06 ^ab^
	Glutamate	6.37 ± 0.13 ^a^	6.40 ± 0.04 ^a^	7.48 ± 0.11 ^b^	9.19 ± 0.11 ^c^
	Proline	2.17 ± 0.10 ^a^	2.31 ± 0.02 ^b^	2.85 ± 0.02 ^c^	3.66 ± 0.05 ^d^
	Glycine	3.10 ± 0.11 b^c^	2.80 ± 0.01 ^a^	2.98 ± 0.03 ^b^	3.24 ± 0.02 ^c^
	Alanine	2.44 ± 0.05 ^a^	2.41 ± 0.05 ^a^	2.71 ± 0.09 ^b^	3.70 ± 0.05 ^c^
	Cysteine	0.51 ± 0.05 ^a^	0.45 ± 0.06 ^ab^	0.39 ± 0.02 ^b^	0.35 ± 0.02 ^b^
	Tyrosine	2.60 ± 0.07 ^a^	2.29 ± 0.07 ^b^	2.35 ± 0.03 ^b^	2.54 ± 0.04 ^a^
	Histidine	1.80 ± 0.09 ^a^	1.55 ± 0.02 ^b^	1.62 ± 0.04 ^b^	1.66 ± 0.07 ^ab^
	Arginine	2.49 ± 0.11 ^a^	2.29 ± 0.03 ^b^	2.51 ± 0.06 ^a^	2.74 ± 0.04 ^c^
	Subtotal	28.53 ± 0.44 ^a^	27.42 ± 0.21 ^b^	30.74 ± 0.17 ^c^	36.05 ± 0.29 ^d^
**EEA (%)**	Threonine	2.18 ± 0.06 ^a^	2.03 ± 0.02 ^b^	2.13 ± 0.05 ^ab^	2.41 ± 0.03 ^c^
	Valine	3.43 ± 0.07 ^ab^	3.22 ± 0.05 ^a^	3.35 ± 0.06 ^ab^	3.65 ± 0.04 ^c^
	Methionine	1.51 ± 0.11 ^b^	1.32 ± 0.03 ^a^	1.23 ± 0.02 ^a^	1.21 ± 0.04 ^a^
	Isoleucine	2.35 ± 0.07 ^a^	2.22 ± 0.03 ^b^	2.41 ± 0.01 ^a^	2.63 ± 0.02 ^c^
	Leucine	2.42 ± 0.08 ^a^	2.32 ± 0.05 ^a^	2.63 ± 0.07 ^b^	3.24 ± 0.05 ^c^
	Phenylalanine	2.99 ± 0.12 ^a^	2.67 ± 0.04 ^b^	2.51 ± 0.02 ^b^	2.69 ± 0.07 ^b^
	Lysine	3.91 ± 0.08 ^b^	3.34 ± 0.07 ^a^	3.27 ± 0.03 ^a^	3.37 ± 0.09 ^a^
	Tryptophan	0.56 ± 0.05 ^a^	0.45 ± 0.02 ^b^	0.42 ± 0.03 ^b^	0.31 ± 0.01 ^c^
	Subtotal	20.35 ± 0.17 ^a^	17.55 ± 0.12 ^b^	17.79 ± 0.14 ^b^	19.51 ± 0.21 ^c^
**Total (%)**		48.88 ± 0.49 ^a^	44.97 ± 0.41 ^b^	48.53 ± 0.36 ^a^	55.71 ± 0.51 ^c^
**EAA/Total**		0.41	0.39	0.37	0.35
**EAAI**		0.98	0.97	0.96	0.92

NEAA, non-essential amino acid; EAA, essential amino acid; EAAI, essential amino acid index. Data are presented as the mean ± SD. Means with different superscripts in column differ significantly (*p* ≤ 0.05). *n* = 3.

## Data Availability

Data is contained within the article.

## Abbreviations

FAO: Food and Agriculture Organization of the United Nations; HPLC, High Performance Liquid Chromatography; PDA, Photodiode array. EAAI, Essential Amino Acid Index; USDA, United States Department of Agriculture; SP, *S. hepiali* polysaccharides; OP, *O. sinens* polysaccharide; SFA, Saturated Fatty Acids; UFA, Unsaturated Fatty Acids; TCA, Tricarboxylic Acid; EAA, Essential Amino Acids; NEAA, Non-essential Amino Acids; WHO, World Health Organization; UNU, United Nations University.
